# Comparison of Multiple NIR Instruments for the Quantitative Evaluation of Grape Seed and Other Polyphenolic Extracts with High Chemical Similarities

**DOI:** 10.3390/foods13244164

**Published:** 2024-12-23

**Authors:** Matyas Lukacs, Flora Vitalis, Adrienn Bardos, Judit Tormási, Krzysztof B. Bec, Justyna Grabska, Zoltan Gillay, Rita A. Tömösközi-Farkas, László Abrankó, Donatella Albanese, Francesca Malvano, Christian W. Huck, Zoltan Kovacs

**Affiliations:** 1 Department of Food Measurement and Process Control, Institute of Food Science and Technology, Hungarian University of Agriculture and Life Sciences, 1118 Budapest, Hungary; lukacs.matyas.krisztian@phd.uni-mate.hu (M.L.); vitalis.flora@uni-mate.hu (F.V.); adrib1196@gmail.com (A.B.); gillay.zoltan@uni-mate.hu (Z.G.); 2 Department of Food Chemistry and Analytics, Institute of Food Science and Technology, Hungarian University of Agriculture and Life Sciences, 1118 Budapest, Hungary; tormasi.judit@uni-mate.hu (J.T.); tomoskozine.farkas.rita.adel@uni-mate.hu (R.A.T.-F.); abranko.laszlo.peter@uni-mate.hu (L.A.); 3 Institute of Analytical Chemistry and Radiochemistry, CCB-Center for Chemistry and Biomedicine, Leopold Franzens University, Innrain 80/82, 6020 Innsbruck, Austria; krzysztof.bec@uibk.ac.at (K.B.B.); justyna.grabska@uibk.ac.at (J.G.); christian.w.huck@uibk.ac.at (C.W.H.); 4 Department of Industrial Engineering, University of Salerno, 84084 Salerno, Italy; dalbanese@unisa.it (D.A.); fmalvano@unisa.it (F.M.)

**Keywords:** dietary supplement, grape seed extract, chemometrics, handheld device, near-infrared spectroscopy, machine learning

## Abstract

Grape seed extract (GSE), one of the world’s bestselling dietary supplements, is prone to frequent adulteration with chemically similar compounds. These frauds can go unnoticed within the supply chain due to the use of unspecific standard analytical methods for quality control. This research aims to develop a near-infrared spectroscopy (NIRS) method for the rapid and non-destructive quantitative evaluation of GSE powder in the presence of multiple additives. Samples were prepared by mixing GSE with pine bark extract (PBE) and green tea extract (GTE) on different levels between 0.5 and 13% in singular and dual combinations. Measurements were performed with a desktop and three different handheld devices for performance comparison. Following spectral pretreatment, partial least squares regression (PLSR) and support vector regression (SVR)-based quantitative models were built to predict extract concentrations and various chemical parameters. Cross- and external-validated models could reach a minimum *R*^2^_p_ value of 0.99 and maximum RMSEP of 0.27% for the prediction of extract concentrations using benchtop data, while models based on handheld data could reach comparably good results, especially for GTE, caffeic acid and procyanidin content prediction. This research shows the potential applicability of NIRS coupled with chemometrics as an alternate, rapid and accurate quality evaluation tool for GSE-based supplement mixtures.

## 1. Introduction

One consequence of the recent globalization of the food market and the vast amount of constantly available food products is the emergence of selective diets with health-preserving aims. Dietary supplements are products designed for consumption with an aim to enhance specific elements of the diet. These products (inexhaustibly) include vitamins, minerals, amino acids, herbs and other botanical derivatives such as concentrates or extracts [1]. Individuals may consume dietary supplements for a variety of reasons; the most prominent motivations include maintaining adequate nutrition, preventing age-related conditions, and safeguarding body tissues from damage. These supplements are often considered a convenient way to address nutritional deficiencies, enhance overall health, and support specific bodily functions, such as immune system health or joint protection, particularly as people age [2,3]. Consumers are presented with an overwhelming array of different products, brands, and formulations distributed through various marketing channels. The dietary supplement market was estimated at USD 177.5 billion in 2023 and is predicted to nearly double by 2030 [4]—the continuous growth in sales implies that these products are becoming an integral part of people’s diets [5].

Polyphenolic compounds derived from grapes have been associated with the prevention of various diseases, including cancer, cardiovascular and neurodegenerative diseases such as Alzheimer’s [6,7,8]. The abundant by-product of the wine industry, grape seeds, is considered an extremely high source of biologically active compounds, which are often further condensed by applying extraction and drying processes to create grape seed extracts (GSE) [9,10]. These extracts are primarily marketed as dietary supplements and are available in forms such as powder mixtures, tablets or capsules.

Serious concerns have been raised by the US Food and Drug Administration (FDA) regarding the safety, efficacy and quality of dietary supplements, especially for those of plant origin, voicing a high risk of adulteration, contamination and the potential absence of key bioactive compounds. These issues are exacerbated by the lack of compositional standardization, leading to batch-to-batch inconsistencies, making it difficult to ensure uniformity in quality and efficacy [11,12]. Furthermore, the compounds themselves are often insufficiently characterized, and there is also a lack of specific quality control methods capable of reliably identifying and quantifying their presence in dietary supplements. Current industrial practices generally rely on unspecific spectroscopic methods, like total phenolic content (TPC) determination and intentional adulteration with organoleptically and chemically similar but cheaper and more readily available substitute compounds, which may go unnoticed in the supply chain [9].

Adding to the significant variability in chemical composition, Villani and colleagues [10], using a high-performance liquid chromatography (HPLC)-based method, found that 9 out of 21 commercially available GSE-based supplements were adulterated with peanut skin extract (PSE)—a cheaper compound with less biological activity and a potential allergen risk. The bioavailability of polyphenols varies significantly between different types, meaning that the most abundant polyphenols in our diet do not always result in the highest concentrations of active metabolites in target tissues [13]. Even among proanthocyanidins, the primary phenolic compounds in GSE with proposed health-promoting benefits, bioavailability greatly varies based on the degree of polymerization, with multiple studies highlighting that only monomers and dimers are absorbed in human intestines in a significant amount [13,14,15]. Peanut skin extract has a similar total phenolic content and antioxidant capacity while mainly consisting of the same proanthocyanidins as GSE, with the difference of containing more of the polymeric fraction with inferior biological activity [10,16]. Differences have also been observed in the bonding patterns of monomers composing proanthocyanidins in GSE and PSE. The former mainly consists of singular bonds (B-type), while the latter consists of double bonds (A-type), generally making the hydroxyl groups of GSE proanthocyanidins more likely to react to and interact with other molecules [17].

Pine bark extract (PBE) is another common adulterant with a high chemical similarity to GSE, containing the same B-type proanthocyanidins (procyanidins with higher polymerization and monomers) as GSE. The only reported differences are the presence of low levels of A-type proanthocyanidins and a relatively higher concentration of biologically inactive polymers [10,16]. While some studies suggest the complete absence of A-type proanthocyanidins in GSE, proposing a potential biomarker to reliably detect PSE and PBE-based adulteration, other research contradicts this, unveiling the presence of type-A proanthocyanidins in certain grape seed varieties [18].

Green tea extract (GTE), although not considered an adulterant of GSE due to its highly active phenolic composition (mainly gallic acid and proanthocyanidin monomers) and generally high value [19,20], is frequently combined with GSE for positive synergistic effects [21]. Food authentication refers to the process of verifying that the product’s ingredients align with its label descriptions and claims [22], and the lack or insufficient amount of valuable and active additives, like GTE, is also a form of consumer deception.

Due to these characteristics, detecting the mentioned additives in GSE is nigh impossible using conventional analytical approaches such as TPC, antioxidant or even total procyanidin assays without the addition of in-depth proanthocyanidin profiling based on sophisticated, costly and time-consuming methods [9]. Additionally, solely focusing on detecting A-type proanthocyanidins might not be sufficient to reliably identify PSE and PBE-based adulteration in GSE. In contrast, non-targeted (fingerprinting) methods, where samples are evaluated by their broad physicochemical characteristics rather than a single biomarker, have proven to be a more realistic approach to identifying these types of fraud [23]. A wide variety of methods can be categorized as non-targeted, with the most significant being various “-omics” disciplines (such as genomics, metabolomics, proteomics, etc.), as well as chromatographic, spectrometric, spectroscopic, and multisensory techniques [24,25]. The speed, non-destructive and non-chemical nature of the measurements and their practical applicability describe the potential application of a vibrational spectroscopy technique to the above-detailed problem.

Near-infrared spectroscopy (NIRS) is among the most commonly applied and rapidly evolving analytical techniques to evaluate food commodities in various industrial settings [26,27,28]. NIRS instruments typically operate based on the Beer–Lambert law, covering a wavelength range of 800 to 2500 nm, though specific spectral intervals and features may differ by instrument manufacturer [29]. The technique measures the absorption intensities as NIR light passes through a sample, which information is then captured, subtracted and correlated to physical or chemical characteristics using chemometric methods. Technologies used to capture NIR absorption spectra vary, with the most conventional NIRS systems using grating or prism setups to split spectra into the desired wavelengths. Digital Light Processing (DLP)-based spectrometers, on the other hand, use a digital micromirror device (DMD) and a single-point detector for wavelength selection, which makes them more suitable for portable designs compared to spectrometers with traditional linear array detectors [30].

Miniaturized vibrational spectroscopy devices enable on-site and real-time assessments of food quality and production processes in the food industry. They have been employed for tasks such as authenticating food products [31,32], monitoring quality changes over time [33,34] and distinguishing foods based on geographical origin [35], indicating the industrial relevance of cheaper and more compact spectroscopic devices. A combination of NIRS and chemometric tools has been used in several cases to investigate certain characteristics of grape seeds, with research successfully detecting grape seed oil adulteration with lower quality edible oils [36] and distinguishing vineyards of origin based on grape skin and seed [37], as well as multiple studies on the accurate prediction of flavonoid compounds in grape seeds [38,39,40].

The benefits of NIR technology can also be observed in the quality control of food supplements produced using plant extracts. Deconinck and colleagues [41] investigated the detectability of different active pharmaceutical ingredients dosed with a series of plant-based supplements using NIRS. Georgieva and colleagues [42] characterized fresh and stored wild berry extracts (bilberry, cranberry, raspberry, strawberry); Gardana and colleagues [43] developed a NIRS-based method for the routine quality assessment of authentic and adulterated bilberry extracts, commonly found in commercial nutritional supplements. Walkowiak and colleagues [44] reported classification accuracies above 87% when detecting Ginkgo biloba leaf extract adulteration with kaempferol, quercetin and rutin in supplements. NIRS also expands the industrial application in the control (i.e., authentication and qualification) of targeted compounds in plants used in manufacturing dietary supplements. This may be particularly important for the detection of regulated plant derivatives containing compounds that may have a toxic effect on the consumer, in addition to reducing product quality, for example, in the case of traditional medicines and nutraceuticals [45,46].

The overview highlights the extensive use of NIRS both in academic and industrial applications. Nevertheless, to the best of our knowledge, there are no published scholarly reports on the detectability and predictability of proanthocyanidin-based adulteration or fortification in grape seed extracts. Detecting such additions is challenging due to the highly similar proanthocyanidin profiles of the frequently used compounds. Addressing this gap, this study aims to develop an NIRS-based method combined with chemometrics to quantify pine bark and green tea extracts in GSE. Additionally, the study evaluates the performance of benchtop and multiple portable NIRS devices, as well as various predictive algorithms, to thoroughly assess the feasibility of the technique in differentiating compounds with high chemical similarities.

## 2. Materials and Methods

### 2.1. Samples and Their Preparation

Authentic grape seed (GSE), pine bark (PBE) and green tea (GTE) extracts were obtained from Xi’an Rongsheng Biotechnology Co., Ltd. (Shaanxi, China) in powdered form. The authenticity of the materials was double-checked using an HPLC method described by Villani et al. (2015) [10]. GSE was mixed with PBE and GTE to formulate singular mixtures, where only a single additive was used (PBMIX, GTMIX), and dual mixtures (PBMIX + GTMIX), where both additives were mixed to GSE simultaneously. Ten additive levels were prepared (0.5, 1, 1.5, 2, 3, 5, 7, 9, 11, 13 *w*/*w*%) this way, with the individual concentrations of added extracts in dual mixtures halved (e.g., 0.25–0.25 *w*/*w*% of PB–GT for level 1, 0.5–0.5 *w*/*w*% for level 2, etc.). The concentration range was selected to align with the industrial practice of adulteration and fortification of these compounds. Samples containing only the pure compounds (GSE, PBE, GTE) were also prepared. Pure GSE samples were prepared in 10 replicates, while all other samples were in triplicates, resulting in a total of 147 samples. All samples were thoroughly homogenized in plastic sample holders.

### 2.2. Methods for the Characterization of Grape Seed Extract Adulteration and Fortification

**Figure 1:**
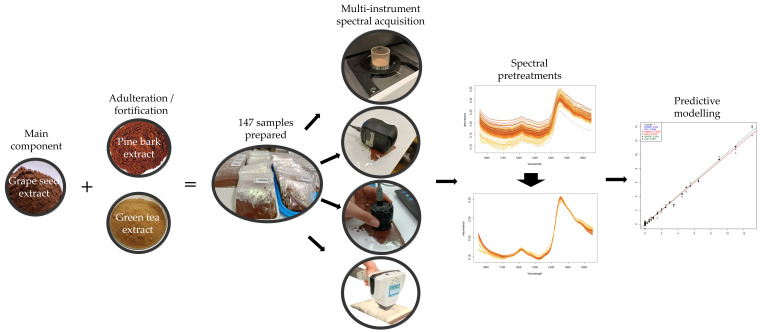
Schematic diagram of the study summarizing sample preparation, spectral acquisition and predictive modeling.

Sample preparation, spectral acquisition and modeling are briefly summarized in Figure 1 and discussed in detail in the following subsections.

#### 2.2.1. Procyanidin Content Determination

For the procyanidin measurement, the method of Porter et al. [47] was adopted. First, 5 mg of the samples was dissolved in 10 mL of methanol (≥99.8% purity). Subsequently, methanolic extracts were diluted to 1:2, 2:5, 3:10 and 1:5. From each of the diluted samples and the original sample, an amount of 1 mL was transferred into 8 mL glass vials, then 6 mL BuOH-HCI solution (95:5) and 200 µL NH_4_Fe(SO_4_)2 × 12 H_2_O solution (2 *w*/*v*%) were added. After mixing, the vials were placed into a water bath and kept at 95 °C for 40 min. The absorbance of the solutions was then measured at 550 nm using a Thermo Electron Nicolet Evolution 300 UV-Vis spectrophotometer (Waltham, MA, USA). All samples were prepared in triplicate.

For the calibration, a condensed tannin extract (Silvateam Welltan, quebracho tree extract (*Schinopsis lorentzii*) 95%) was used in the 0.02–0.2 mg/mL concentration range. Based on the calibration curve, the results were calculated in mg (condensed tannin equivalent) per mL of extract (mg/mL). Reagents were acquired from VWR International Kft., Debrecen, Hungary.

#### 2.2.2. Antioxidant Capacity Determination

For antioxidant activity (AA) determination, the 2,2-diphenil-1-picrylhydrazil (DPPH) radical scavenging assay was used [48].

An amount of 0.5 g of GTE, GSE and PBE samples were dissolved in 5 mL of methanol and stirred for 30 min. Successively, 134 µL of each sample solution was added in a cuvette containing 3.9 mL of a 6 ∗ 10^−5^ M methanol solution of DPPH. The cuvette was incubated in the dark for 40 min at room temperature. The bleaching of DPPH was recorded at 515 nm by a UV–6300PC Double Beam Spectrophotometer (VWR International S.r.l., Italy) at room temperature (A_sample_). A blank experiment was also carried out by applying the same procedure to a solution without sample (A*_blank_*). The antioxidant activity was expressed as the percentage inhibition of DPPH and calculated as follows:

$$\%\;inhibition\;of\;DPPH=\frac{\left(A_{blank}-A_{sample}\right)}{A_{blank}}*100$$

Antioxidant activity was finally expressed as Trolox equivalent. Values were determined by a calibration curve of Trolox standard solutions (60–600 µM), performed according to sample preparation. The results were expressed as µmol of Trolox equivalent per gram of sample. Reagents were acquired from VWR International S.r.l., Milan, Italy.

#### 2.2.3. HPLC Method for Proanthocyanidin Monomers (Gallic Acid, Catechin, Epicatechin) and Caffeic Acid Determination

HPLC analysis was performed using a Hewlett Packard HP-1100 series (Palo Alto, CA, USA) fitted with an auto sampler and a diode-array detector (DAD). The separation was achieved on a Poroshell 120 EC-C18 column (150 × 4.6 mm I.D., 4 μm particle size) (Agilent, Santa Clara, CA, USA). The column temperature was set at 25 °C, and the injection volume was 2 μL. The mobile phase was used in gradient mode. The elution was performed using 1% acetic acid in acetonitrile (*v*/*v*) (A) and 1% acetonitrile in water (B), and the flow rate was set at 1.2 mL/min. HPLC/DAD analyses were performed monitoring at 280, 310 and 350 nm. Phenolic compounds were identified by comparing retention time and UV absorption spectra with available standards. Quantification was performed with standard curves of external standards generated by plotting HPLC peak areas against the concentrations (mg/L). Final values were expressed in mg/g. Reagents were acquired from VWR International S.r.l., Italy.

#### 2.2.4. NIR Spectral Acquisition

One benchtop and three handheld/portable NIR spectrophotometers were used for spectral acquisition. NIR devices were selected to provide multiple comparison options, including spectra acquisition technology, recording ranges and resolution.

The NIRS XDS (Metrohm, Glostrup, Denmark) with the RapidContent Analyzer module was used as the benchtop device. The device operates with a dispersive grating monochromator to acquire spectra in the 400–2500 nm wavelength range at 0.5 nm intervals. Diffuse reflectance signals were collected by a silicon (Si) detector in the 400–1099.5 nm range and a lead sulfide (PbS) detector in the 1100–2500 nm range.

Regarding the handheld instruments, the NIR-S-G1 (InnoSpectra Co., Hsinchu, Taiwan) with a DLP micromirror array and an InGaAs detector was used in the wavelength range of 900–1700 nm and a spectral resolution of 3 nm. The second handheld instrument was the MicroNIR 1700 EC (Viavi Solutions Inc., Chandler, AR, USA), featuring a multielement InGaAs array detector paired with a linear variable filter (LVF). Spectra were collected in diffuse reflectance mode in the 908–1676 nm wavelength spectral range with a resolution of 6.2 nm. The microPHAZIR (Thermo Fisher Scientific, Waltham, MA, USA), a fully autonomous handheld instrument with its own built-in user interface, was the third portable device. The instrument uses a micro-optoelectro-mechanical system (MOEMS) for wavelength selection and an InGaAs detector; it provides a spectral resolution of 12 nm (optical) in the measured range of 1596–2396 nm.

With the benchtop device, sample spectra were collected through an optical glass window cuvette. To minimize variations in light scattering, the powdered samples were compacted uniformly by gently tapping the cuvette three times on a laboratory workbench before collecting the spectra. Due to previous findings unveiling a potential better performance for scanning samples through plastic bag containers compared to using cuvettes in the case of DLP-based devices [49], all handheld data were collected by scanning samples directly in low-density polyethylene (LDPE) bags. Additionally, this approach was also intended to better simulate the industrial application of portable devices.

All spectra were collected at room temperature (25 °C) with 3 consecutive scans, resulting in a total of 1764 measurements across all samples and spectrometers. Both temperature and humidity levels during spectral acquisition were monitored using a Voltcraft DL-121TH multi-data logger (Conrad Electronic, Berlin, Germany) to account for any substantial environmental condition.

### 2.3. Statistical Methods

#### 2.3.1. Univariate Statistical Comparison of the Chemical Reference Results

One-way ANOVA was used to identify significant differences between the chemical measurement values obtained for each raw extract. Prior to this step, the validity of the input data for ANOVA was ensured; homogeneity of variances was verified by Bartlett’s test, whereas the Shapiro–Wilk test was used to check for normality. Homogeneity of variances and normality could not be accepted when comparing gallic acid (K(2) = 13.99, *p* < 0.01; W = 0.76, *p* < 0.01) and catechin values (K(2) = 10.73, *p* < 0.01; W = 0.8, *p* < 0.05) of the different extracts even after the evaluation of skewness and kurtosis; hence, these parameters were left out of univariate statistical analysis. For the remaining chemical parameters (procyanidin content, antioxidant capacity, caffeic acid, and epicatechin), which passed the assumption tests for ANOVA, pairwise comparisons were made between the extracts using Bonferroni’s post hoc test.

#### 2.3.2. Exploratory Analysis of the NIR Spectroscopy Results

Raw spectra recorded with each spectrometer were visualized to identify prominent absorbance regions and determine the appropriate spectral pretreatment to apply. Averaged raw spectra of the pure extracts and the second derivatives of the raw spectra containing only the highest levels of added extracts were also visualized to identify and match prominent absorbance peaks.

Principal component analysis (PCA) was employed for the inspection of the data structure, pattern recognition, and outlier detection [50]. Principal components were also used as inputs for support vector regression-based modeling. Prior to modeling, various pretreatments were used to reduce baseline variations and slope differences caused by unwanted variance and spectral noise, including Savitzky–Golay (SG) filter with second-order polynomial and varying smoothing points between 11 and 41, standard normal variate (SNV), multiplicative scatter correction (MSC), detrending (deTr), first (FD) and second (SD) derivatives [51]. Optimal combinations were selected by observations on raw spectra and PCA score plots, following further tuning during supervised modeling. In general, the combination that reduced baseline shifts and discrepancies in curvature the most while emphasizing chemically identified prominent absorbance peaks and regions was selected for supervised modeling. Similarly, specific wavelength ranges were also selected for each instrument, focusing on regions identified as containing the most valuable information during the exploratory analysis. During wavelength selection, regions identified as having poor signal-to-noise ratio, extremely high absorbance values or exhibiting spectral artifacts originating from sensor/instrument characteristics were generally omitted.

#### 2.3.3. Predictive Modelling of the NIR Spectroscopy Results

The concentration of each extract (GSE, PBE, GTE), the procyanidin content (proanthocyanidins with ≥2 level of polymerization), the antioxidant activity, the concentration of proanthocyanidin monomers (gallic acid, catechin, epicatechin) and caffeic acid were modeled and predicted using partial least squares regression (PLSR) and support vector regression (SVR). Each model was individually optimized to reach the highest predictive performance while avoiding overoptimistic predictions by model overfitting. The models were cross-validated using leave-three-consecutive-out, and an external validation via a test set was also applied to the most promising models to definitively assess their robustness. For test-set prediction, two-thirds of the data (two replicates) were used for training, and one-third (one replicate) was reserved for testing, repeated three times to have all samples both in the training and the validation set. Alongside the spectral pretreatments in the case of PLSR models, latent variable (LV) numbers were also adjusted to capture enough variance without the risk of overfitting. LV selection was achieved using error plots, model performance metrics and observations on regression vectors [52].

SVR models were optimized by selecting the number of input PCs and by simultaneous hyperparameter tuning. Hyperparameter tuning involved adjusting the error weight (C: 0.1–10) and the maximum error value (ε: 0.01–0.5) while also testing different kernel functions (linear, radial, polynomial, and sigmoid) [53]. The cost function was used to minimize both the model coefficients and prediction errors simultaneously, aiming to achieve the best-performing model for each set of parameters [54] while keeping in mind that excessively large coefficients can reduce the generalization ability of the model by increasing variance, leading to overoptimistic predictions. The risk of model overfitting was reduced by setting a maximum acceptable difference of ~15% between calibration and validation average error values. The difference was followed by scree-plots as the number of components was gradually increased. As a general rule of thumb, the maximum number of components was set not to exceed 1/10th of the total number of samples in the model [55]. For models built with a linear kernel, regression vectors were also visualized and evaluated.

All regression models were evaluated based on model performance metrics, including root mean square error of calibration (RMSEC), root mean square error of cross-validation (RMSECV), root mean square error of test-set prediction (RMSEP), determination coefficient of calibration (*R*^2^_C_), determination coefficient of cross-validation (*R*^2^_CV_) and determination coefficient of test-set prediction (*R*^2^_P_). Material concentration ranges were not selected to specifically test detection limits due to a more profound focus on modeling real industrial fortification/adulteration schemes and hence using higher minimum concentrations than the presumed detection limit of the devices; for the best performing models, the limit of detection (LOD) and quantification (LOQ) values were still calculated for comparative reasons. In general, LOD is defined as the lowest concentration of an analyte in a sample that can detected, but not necessarily quantified, under specific test conditions, while LOQ refers to the minimum concentration of an analyte in a sample that can be measured with sufficient precision and accuracy. LOD and LOQ values were established using a conventional regression-based method assuming no serious background noise [56], which is applicable for both SVR and PLSR models equally. For a linear calibration curve, the instrument response (y) was presumed to have a direct linear relationship with the standard concentration (x) over the defined range of values. This relationship is expressed as follows:

y=a+bx

where *a* represents the intercept, and *b* is the slope indicating sensitivity. Using this model, the LOD and LOQ can be calculated as follows:

$$LOD=3*\frac{Sa}{b}$$

$$LOQ=10*\frac{Sa}{b}$$

where *S*_a_ is the residual standard deviation of a test-set predicted model. All data evaluation and visualization were achieved using R-project (v. 4.3.0, 2023, The R Foundation for Statistical Computing, Vienna, Austria; using R package: aquap2 [57]).

## 3. Results

### 3.1. Chemical Measurement Results of the Extract Mixtures

**Figure 2:**
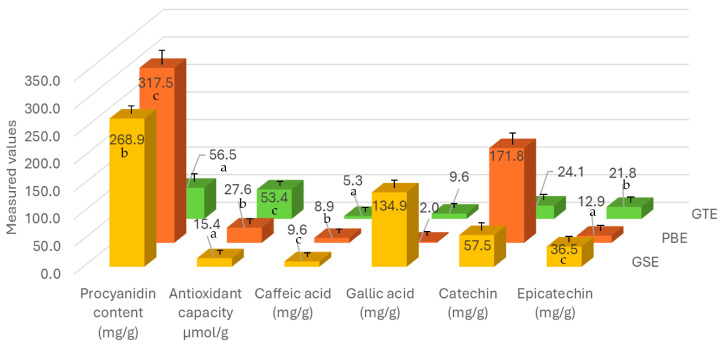
Summary of the averaged chemical measurement results for all three raw extracts (GSE, PBE, GTE). Different letters indicate statistically acceptable significant differences (*p* < 0.01) between extracts.

Chemical measurement results for all three raw extracts are summarized in Figure 2. The highest average procyanidin content was measured for PBE at 317.5 ± 13 mg/g, significantly more (F(8) = 373.4, *p* < 0.01) than in the case of GSE (269 ± 8.1 mg/g) and GTE (56.5 ± 8.6 mg/g). The applied procyanidin assay does not account for monomeric flavan-3-ols, which fraction was previously reported to be much higher in GSE than in PBE [10]. By combining the monomers (gallic acid, catechin, epicatechin), present results are in agreement with this observation, with GSE having a significantly higher (F(2) = 5759, *p* < 0.01) monomeric fraction of 228.9 ± 1.3 mg/g than PBE (186.7 ± 2.6 mg/g) and GTE (55.4 ± 0.4 mg/g). The magnitude of these results is also in agreement with previous findings, where authors reported values between 9.3 and 14% [58] and 162.5 mg/g [10] for the monomeric fraction in GSE. The results can be greatly different if galloylated monomers are also counted. GTE was reported to have the lowest degree of polymerization out of the three extracts in previous studies [16,59], explaining the present low procyanidin (which only counts dimers and up) but comparatively high antioxidant capacity results. The present study does not discuss galloylated monomers, which are by far the most abundant phenolic compounds in GTE–the concentration of epigallocatechin gallate (EGCG) alone reported to reach up to 250–950 mg/g [16], whereas EGCG was also identified as the highest contributing tea polyphenol in antioxidant assays [60]. In alignment with these, for antioxidant capacity, a significantly higher (F(2) = 108.1, *p* < 0.01) value was measured for GTE (53.4 ± 1.9 µmol/g) compared to GSE (15.4 ± 0.4 µmol/g) and PBE (27.6 ± 3.5 µmol/g). A roughly two times higher antioxidant capacity value for GTE compared to PBE was similarly reported previously [20]. The main difference between GSE and PBE was the monomer composition, with GSE showing higher gallic acid and lower catechin values compared to PBE. Whereas these differences could not be statistically proven, they were in agreement with previous research [10]. Caffeic acid, a highly bioavailable polyphenolic monomer [61], was present in roughly one magnitude lower concentration than the prominent monomers in each extract, as was expected based on literature data [62].

### 3.2. Exploratory Data Evaluation of the NIR Spectroscopy Results Based on Raw Spectra and PCA

**Figure 3:**
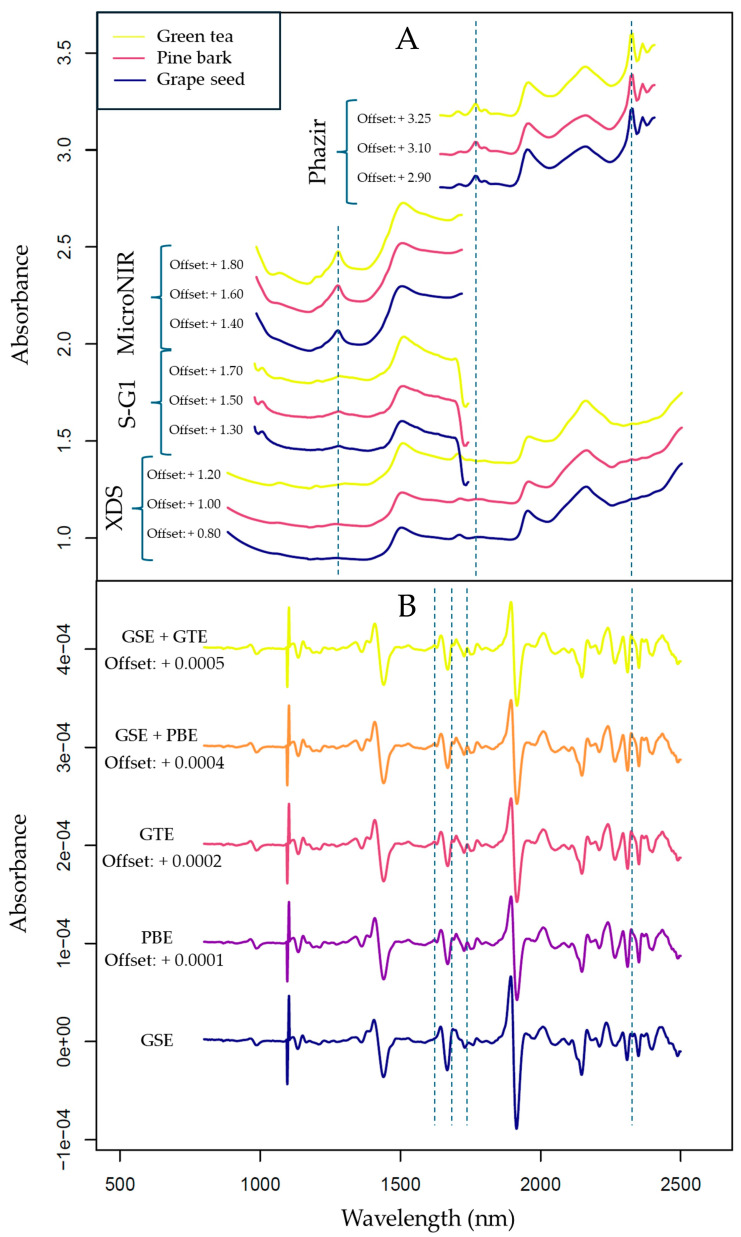
Averaged raw spectra of the extract samples: (**A**) raw spectra of the pure extracts recorded with all four devices; (**B**) second derivative of the raw spectra of pure extracts and samples containing the highest amount of GTE or PBE added, recorded with the benchtop device (XDS). The visible range of the benchtop device (400–799.5 nm) was excluded. Second derivatives were calculated using Savitzky–Golay filter with second-order polynomial and 21 smoothing points. Vertical lines indicate notable peak differences between the spectra of the individual sub-datasets. Vertical offsets provide better interpretability of the plot; these offsets were not introduced during data analysis.

The raw spectra of the measured samples are summarized in Figure 3. Due to the chemical similarities of the extracts, only subtle differences could be identified by visual inspection of the untreated spectra (Figure 3A), whereas there were some visible differences between the spectra recorded by the different spectrometers. Most notably, as the measurements with miniaturized spectrometers were taken directly in the container bags, the presence of LDPE bands could be noticed in the corresponding spectra. These were best manifested in the longer wavelength part of the NIR region that only microPHAZIR measured, i.e., fairly strong and well-resolved peaks at ~2350 nm (binary combinations of CH stretching and CH deformation modes) and at ~1800 nm (first overtones as well as binary combinations of CH stretching modes). The polymer bands towards the short-wavelength boundary of the examined region were weaker; nonetheless, the second overtone of C-H stretching of LDPE could be seen at ~1220 nm in the spectra acquired by MicroNIR (relatively better resolved) as well as NIR-S-G1 (less pronounced in the intensity). The positions and appearance of these peaks are characteristic of PE polymer, regardless of the matrix [63]. Importantly, the presence of the superimposed bands of the bag material did not prevent successful predictions of the constituents of interest in this study, both in the case of handheld instruments operating in the short- and long-wavelength part of the NIR region.

Spectra acquired with the handheld devices, however, showed distorted results near the boundaries of their spectral regions in most cases; hence, these regions were deemed unreliable and were omitted [64,65]. Since the XDS operates with two detectors with a noticeable baseline shift at 1100 nm, the wavelengths below 1100 nm were excluded before further modeling. Based on these observations, the 1100–2250 nm range was selected for the XDS, the 1630–2250 nm range for the microPHAZIR, and the 950–1650 nm range for the NIR-S-G1 and microNIR as the regions potentially containing the most useful information. The spectra recorded with handheld devices showed both a baseline and a slope shift, indicating the necessity to apply spectral correction prior to further analysis. SNV was applied to account for the baseline variance, whereas detrending proved to be effective at mitigating slope differences [66].

By looking at the second derivative of the spectra acquired with the benchtop device (Figure 3B), subtle differences could be observed between the spectra of pure GSE and samples that contained the additives in the highest concentration. These differences were mainly present in the 1650–1750 nm range (the first overtone region), where mainly C-H stretching bands can be found, with the additional possibility of S-H (aromatic) bands also manifesting in there [67]. Polyphenolic compounds, like proanthocyanidins, are known for containing aromatic rings where C-H bonds are present; therefore, this region could explain compositional differences between samples.

**Figure 4:**
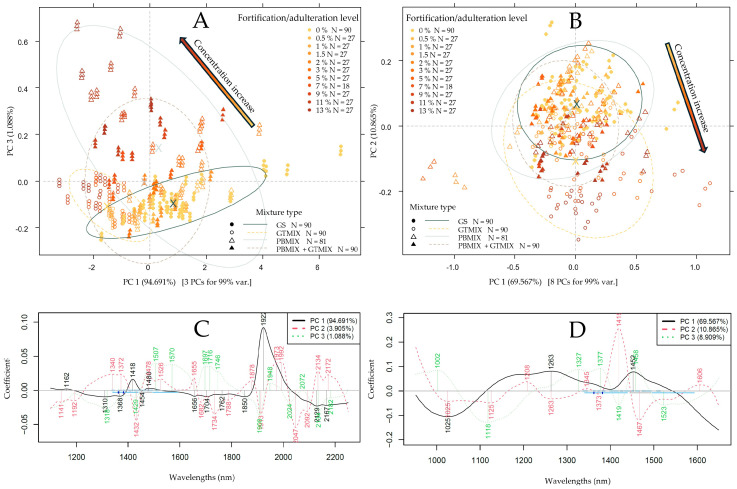
PCA results to separate measurement points based on additive concentration (gold-brown gradient coloring) and mixture types (marker type and ellipses). Savitzky–Golay filter with second-order polynomial and 21 smoothing points, detrending and SNV as pretreatment: (**A**) PCA score plot based on XDS data; (**B**) PCA score plot based on NIR-S-G1 data; (**C**,**D**) corresponding loading vectors.

**Figure 5:**
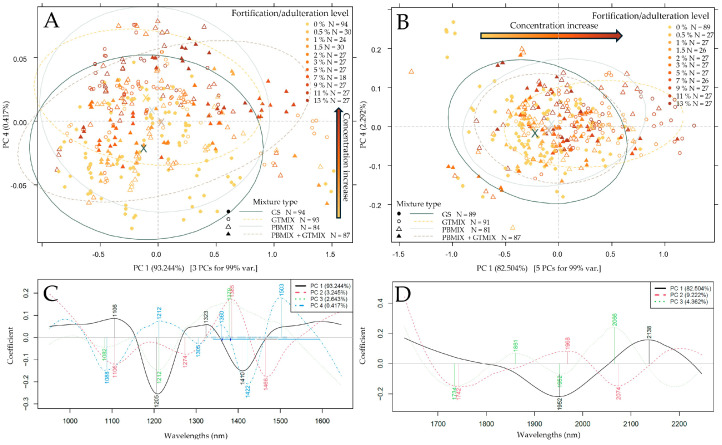
PCA results to separate measurement points based on additive concentration (gold-brown gradient coloring) and mixture types (marker type and ellipses). Savitzky–Golay filter with second-order polynomial and 21 smoothing points, detrending and SNV as pretreatment: (**A**) PCA score plot based on MicroNIR data; (**B**) PCA score plot based on microPHAZIR data; (**C**,**D**) corresponding loading vectors.

Following the initial application of spectral pretreatments and the selection of the prominent regions, the results of PCA were summarized in Figure 4 and Figure 5. In the case of the benchtop device (Figure 4A), the separation of measurement points could mainly be seen along the third principal component. The corresponding loading vectors (Figure 4C) unveiled the most important wavelength regions in this separation to primarily be at ~1700–1750 nm with S-H and C-H stretching bands. Another notable peak at ~1908 nm can predominantly be attributed to moisture and potential O-H stretching vibrations caused by carboxyl groups of (phenol) carboxylic acids [67]. A certain extent of separation alongside PC1 was also visible in the form of inter- and intra-group variance, which could probably be due to the slight differences in moisture content based on the prominent peak at 1922 nm. In the case of the NIR-S-G1, the PCA score plot (Figure 4B) showed the most notable separation of measurement points belonging to different additive concentration values alongside the second principal component, accounting for ~11% of the total variance. By observing the corresponding loading vectors (Figure 4D), the most significant peaks for this separation were at 1419 and 1467 nm. Water molecules are known to have strong absorption at 1400–1450 nm, while aromatic O-H stretching bands are also characteristic of this region, which could be attributed to the presence of phenolic compounds [67]. PCA score plots also indicated the necessity for outlier detection prior to regression modeling.

In the case of the MicroNIR (Figure 5A) and the microPHAZIR (Figure 5B), measurement points belonging to the different additive concentrations and types on PCA score plots show a significant overlap. For the MicroNIR, a notable separation could only be identified along the fourth PC, explaining ~0.4% of the total variance. Prominent peaks could be identified at the overlap of the first and second overtone regions with O-H, (aromatic), C-H and N-H stretching vibrations, which could indicate the presence of organic compounds like carbohydrates, hydrocarbons and proteins. For the microPHAZIR, a separation of points could be seen alongside PC1, holding ~82.5% of the total variance, with two major peaks at ~1952 nm and ~2138 nm on the loadings vector (Figure 5D). These peaks are associated with C-H and O-H and N-H combination bands, and they are also visible on the XDS PCA loadings (Figure 4C) with a slight drift. Overall, PCA results based on the data of all devices show notable separation based on additive levels with a high probability of signaling a change in phenolic compound composition.

### 3.3. Predicting Extract Concentrations and Chemical Parameters Using PLSR and SVR

As anticipated [49,68], the best models for every parameter were built using the benchtop (XDS) dataset. The results of extract concentration prediction using PLSR and SVR are summarized in Table 1 and Table 2, respectively. For the prediction of GSE concentration, the best-performing model using the XDS dataset and PLSR could reach an *R*^2^_CV_ value of 0.99 with an RMSECV value of 0.446%, followed by the MicroNIR and PLSR combination with an *R*^2^_CV_ of 0.767 and an RMSECV of 2.128%. To quantify pine bark-based adulteration, both the PLSR and the SVR models could reach remarkable fit quality using the benchtop dataset with the highest *R*^2^_CV_ value of 0.993 and the lowest RMSECV value of 0.268%. Models built on handheld datasets, however, had poor to medium model fits in predicting PBE concentrations, with the microPHAZIR dataset proving to be the best with an *R*^2^_CV_ of 0.671 and an RMSECV of 1.79%. For the prediction of green tea extract concentration, high predictive performance was reached with almost all dataset-algorithm combinations, with the best model built by combining XDS data with SVR, reaching an *R*^2^_CV_ of 0.985 and an RMSECV of 0.396%. Out of the handheld devices, a comparably good performance was achieved by using the NIR-S-G1 dataset and PLSR with an *R*^2^_CV_ of 0.965 and an RMSECV of 0.630%.

For the prediction of procyanidin, antioxidant capacity and caffeic acid (Table 3 and Table 4), most dataset-algorithm combinations resulted in models with good predictive performance. By far, the best models to predict proanthocyanidin content were achieved using the XDS–SVR and NIR-S-G1–PLSR combinations, with respective *R*^2^_CV_ and RMSECV values of 0.988, 0.805 mg/g and 0.95, 1.629 mg/g. These results are comparable to previous research predicting procyanidin content using NIRS data [69]. For antioxidant capacity prediction, models built with SVR generally performed better than PLSR, with once again the XDS and NIR-S-G1 datasets proving most suitable for model building, reaching respective *R*^2^_CV_ and RMSECV values of 0.983, 0.167 µmol/g and 0.914, 0.373 µmol/g. Comparing the other two devices for antioxidant capacity prediction, the model built on MicroNIR compared to microPHAZIR data was slightly more feasible, in agreement with previous findings [70]. To predict caffeic acid content—similarly to the case of procyanidin content prediction—the XDS–SVR and NIR-S-G1–PLSR combinations provided the best models with respective *R*^2^_CV_ and RMSECV values of 0.982, 0.019 mg/g and 0.951, 0.031 mg/g. In general, models predicting caffeic acid based on NIR-S-G1 and MicroNIR data were close to the performance of the benchtop model, with the microPHAZIR reaching slightly worse model metrics. This implies the significance of the second overtone region in predicting this particular phenolic monomer.

The predictive results for the proanthocyanidin monomers were summarized in Table 5 and Table 6. Compared to the performance of benchtop-based models, models built on handheld device data showed a notably worse performance for all monomers. Out of the handheld datasets, contrary to the observations for caffeic acid, these monomers were predicted with the lowest average error using microPHAZIR data in all cases, indicating the potential significance of the first overtone and combination band regions to predict these compounds. This region was also prominent for the separation on PCA score plots for the XDS and microPHAZIR datasets (Figure 4A and Figure 5B). Gallic acid, the most abundant monomer in GSE, was predicted with an *R*^2^_CV_ of 0.985 and RMSECV of 0.682 mg/g with the XDS-PLSR combination, while the model based on microPHAZIR data could reach an *R*^2^_CV_ of 0.769 and RMSECV of 2.738 mg/g. Outside of gallic acid prediction, SVR provided slightly better models for the XDS datasets, while PLSR was undeniably better at building predictive models for all handheld datasets when predicting proanthocyanidin monomers.

As anticipated, the benchtop spectrometer produced the most information-rich and reliable data, leading to the best-performing predictive models. All the miniaturized instruments examined in this scenario showed measurably lower performance, which must be accepted for on-site applications. Among the handheld instruments, the NIR-S-G1 achieved the best model fits and lowest average errors in most cases. Comparing the regression methods, while SVR provided some of the best-performing models, mainly for the benchtop instrument, PLSR showed an overall better performance across the board, especially in the case of handheld datasets. Interestingly, the available literature presents an inconsistent background for this conclusion; previous studies both align with [71] and contradict [72] the observation that PLSR offers better performance in modeling the relationship between NIR spectral data and polyphenolic reference. When looking at the different parameters, the best model fit for the benchtop device was achieved when predicting PBE concentration, while it was the prediction of GTE concentration for the NIR-S-G1.

**Figure 6:**
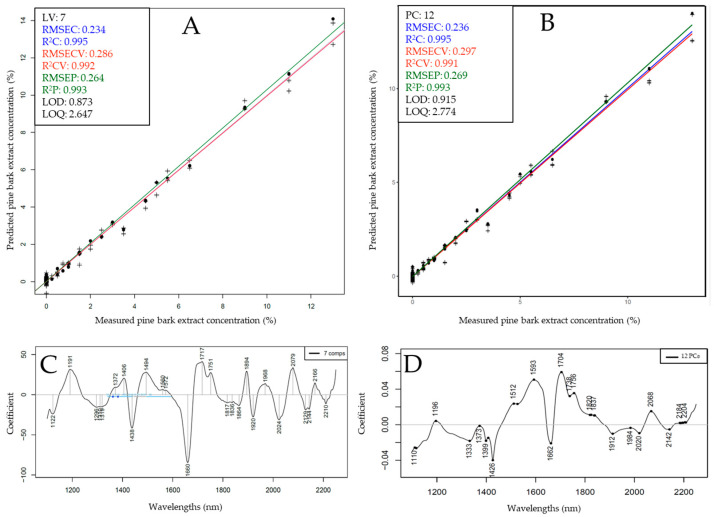
Test-set prediction results based on benchtop (XDS) data to predict pine bark extract concentration: (**A**) Y-fit plot using PLSR; (**B**) Y-fit plot using SVR; (**C**,**D**) corresponding regression vectors.

**Figure 7:**
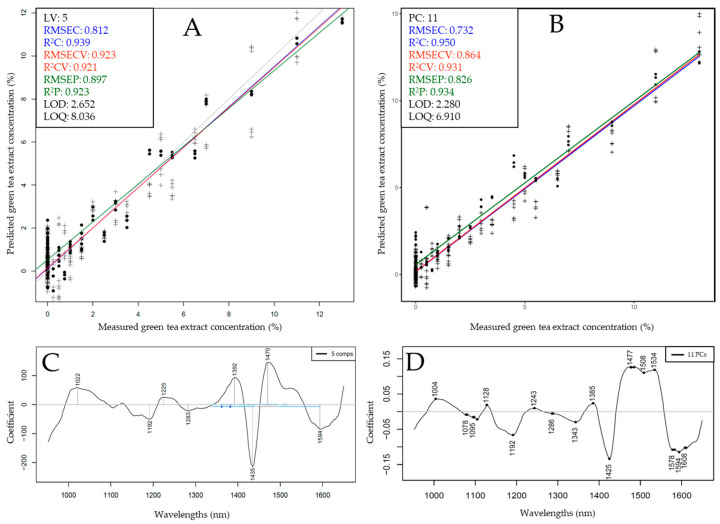
Test-set prediction results based on handheld (NIR-S-G1) data to predict green tea extract concentration: (**A**) Y-fit plot using PLSR; (**B**) Y-fit plot using SVR; (**C**,**D**) corresponding regression vectors.

For these reasons, test-set prediction was performed for the XDS and NIR-S-G1 for the prediction of PBE and GTE, respectively, while using both PLSR and SVR as algorithms. The results of test-set prediction are shown in Figure 6 and Figure 7.

Following the test-set split, pine bark extract concentration could be predicted with almost equally good model metrics using PLSR and SVR, with PLSR models exhibiting slightly lower average error values, resulting in an *R*^2^_P_ of 0.993 and an RMSEP of 0.264%. A slightly lower LOD and LOQ value was also observed for the PLSR model at 0.873% and 2.647%, respectively. Upon inspecting the regression vectors (Figure 6C,D), several prominent peaks could be identified throughout the entire recorded spectral range, with the highest intensity bands visible at the intersection of the first and second overtone region between ~1370 and 1750 nm. The most notable peaks in this region could correspond to (aromatic) hydrocarbons and (aromatic) hydroxyl groups, which are strong indicators of the presence of phenolic compounds. Other significant peaks were visible in the ~2060–2080 nm wavelength range of the combination band region, which area also corresponds to the vibration of hydroxyl groups, either aliphatic or phenolic. These observations are in good agreement with the PCA loadings (Figure 4C), where measurement points belonging to the different additive concentrations were separated based on these same regions.

Test-set validated models built to predict green tea extract concentration with the NIR-S-G1 dataset reached slightly better performance metrics when using SVR compared to PLSR (Figure 7). The best-performing model could reach an R^2^_P_ of 0.934 and RMSEP of 0.826% with a corresponding LOD value of 2.28% and LOQ value of 6.91%. A roughly 3 times higher LOD and LOQ value between benchtop and handheld NIR spectrometers was also reported in a similarly designed recent study [49]. Upon comparing PLSR and SVR regression vectors (Figure 7C,D), although with a slight drift, the same prominent bands were visible with the strongest contribution of the region in the overlap of the first and second overtones, more specifically between ~1380 and 1600 nm. These peaks can be identified on the PCA loadings vectors when separating measurement points along the increase in additive concentration (Figure 4D) and on the regression vectors corresponding to models based on benchtop data (Figure 6C,D). Test-set validation and calibration average errors in the case of all models were less than 15% apart, while the identical observations on loadings and regression vectors across different devices and algorithms imply robust model fits, presumably capturing the chemical differences between samples.

## 4. Discussion

NIRS offers multiplexing analytical capacity, i.e., the absorption characteristics of sample results from the co-additive contribution of all its chemical constituents rather than solely focusing on any specific one of them. Consequently, it can effectively identify foreign substances in chemically similar matrices even without selecting a biomarker, as demonstrated in this and previous research [49,53,68,73]. This is especially true for matrices where only subtle differences can be identified, but between multiple chemical constituents at once. The scientific literature available for direct comparison of the present results is very scarce. Studies presenting chemical measurements for extracts in focus here show great variability both for the exact chemical profile and the concentration ranges. Villani and colleagues (2015) [10] measured an average of 383.5 mg/g proanthocyanidin content (involving monomers) in GSE, supporting present findings (497.8 mg/g, combining monomers with procyanidin result), whereas other researchers have reported values much higher, around 900 mg/g measured with multiple methods [58]. This is also true for PBE, where there is a report of total proanthocyanidin values as low as 64.4 mg/g [10] and as high as 883 mg/g [20]. Values can greatly differ based on the exact way a measurement method is applied and the variety of the plant from which the extracts are derived. For instance, Masson pine bark extracts are reported to be 5–10 times cheaper than extracts of maritime pine bark, with a vastly different ratio of proanthocyanidin fractions [16]. The present results, which demonstrate that PBE has a higher antioxidant capacity (27.6 ± 3.5 µmol/g) compared to GSE (15.4 ± 0.4 µmol/g), are generally contradicted by previous findings, where GSE was reported to have 10–30% higher antioxidant values compared to PBE [10,74]. The different results could be attributed to the different PBE variants used, as well as the proposed high sensitivity of the DPPH assay to catechin over gallic acid. Both similar [60] and higher [75] radical scavenging activities for catechin compared to gallic acid have been reported before. This is logical from a structural point of view, as catechin, a more complex molecule, has more hydroxyl groups to donate hydrogen atoms from to contribute to radical scavenging. On a side note, in vitro assays do not account for absorption and bioavailability, where gallic acid has been reported to significantly outperform other, more complex phenolic compounds [76,77].

The structural similarities and differences of these molecules could also explain the results of NIR modeling. The overall lowest predictive performances were reached in the case of catechin and epicatechin, presumably due to the similarity of these compounds to each other and to the fact that procyanidins are also mainly built up by these molecules. This could explain the generally poor performance of models built on handheld device data to predict these two monomers. Among other technical differences, their inferior spectral resolution is most likely the primary limiting factor, as well as a narrow wavelength range to characterize these subtle chemical variances. The present results also pointed out that the first overtone and combination band regions are presumably the most important to characterize proanthocyanidin monomers, whereas procyanidin concentrations can be well predicted just by using the first overtone region for spectral acquisition, generally showing promising results with handheld devices covering this region. However, the performance of this analysis seems to be matrix-dependent and requires a case-to-case feasibility screening, as previous research has reported high predictive accuracies for catechin (*R*^2^ = 0.962) and epicatechin (*R*^2^ = 0.942) using the same DLP-based handheld device when investigating instantized green tea samples [78]. Gallic acid, the most abundant monomer in GSE with more distinct structural characteristics, was generally predicted with better metrics in the present study. These results, however, are still slightly inferior to previous research predicting gallic acid concentration in black tea samples with a handheld device covering a similar spectral range and PLSR, reaching an *R*^2^ of 0.865 and RMSECV of 4.94 mg/100g [79]. These differences (among many other factors) could be attributed to the more complex matrices used in the present study, highlighting the heavy matrix dependency of the technique [80]. Caffeic acid, on the other hand, despite its low concentration and small differences between samples, was very well predicted with most handheld devices, probably due to its more distinct structure compared to the rest of the molecules in the matrix. These results were comparable to a previous study, where the authors used a handheld device covering the same 900–1700 nm spectral range to predict caffeic acid content in jujube, reporting an *R*^2^ of 0.878 and an RMSECV of 6.79 μg/g [81].

While predicting the chemical parameters is important to characterize these matrices, the present study focused more on the detection and quantification of the extracts as a whole. While there are several studies using (portable) NIRS in combination with chemometrics to accurately quantify various types of proanthocyanidins in different matrices [64,69,70,78,79,81,82], to the authors’ best knowledge, no attempts were made so far to characterize GSE adulteration/fortification using the technique before. A general observation could be made that extracts with higher chemical similarities (GSE, PBE) were more difficult to predict based on handheld data but not based on the benchtop data with better spectral resolution and wavelength range, potentially capturing enough of the variance among samples. Out of the handheld devices, the microPHAZIR, operating in the first overtone and combination band region of the NIR spectrum, was more feasible to capture the variance differentiating these two extracts, much like in the case of the prominent proanthocyanidin monomers building them up. Green tea extract, due to its more distinct proanthocyanidin composition, was much better predicted with all devices, with the NIR-S-G1, a device limited mainly to the second overtone region, contributing to models nearly as feasible as the benchtop device, even when a test-set was applied. Once again, this observation was in agreement with the chemical composition prediction, where models based on NIR-S-G1 and MicroNIR data had better overall performance.

It should be noted that the measurements using the benchtop spectrometer corresponded to the idealized setting representative for a typical in-lab analysis. This means better-controlled sample presentation using cuvettes that lead to uniformly compacted samples, which reduces variability in light scattering. Handhelds measured samples through LDPE bags, which introduces a degree of variability due to the absorbance of the container material and sample packing, which influences the scattering and reflectance properties. These effects did not prevent a successful analysis with the handhelds, and the examined approach is a perfect representation of a practical application scenario in which rapid, through-bag scanning directly on-site (i.e., independent from the laboratory, mitigating any need for sample preparation) delivers a breakthrough advantage. That being said, as discussed earlier, none of the examined handheld instruments could deliver consistently superior results among all analytical properties in consideration here.

A closer inspection of the predictive performance of the miniaturized spectrometers generally shows that all the analyses in focus here can be reliably performed with these instruments. However, the consistency of the performance among the compared handheld spectrometers is not as great as it is for the benchtop one. With consistency in mind, the NIR-S-G1 tends to perform the best, particularly in PLSR for procyanidin content (*R*^2^_CV_ = 0.950, RMSECV = 1.629 mg/g) and antioxidant capacity (*R*^2^_CV_ = 0.907, RMSECV = 0.373 µmol/g). It also outperforms other handhelds in green tea extract prediction (*R*^2^_CV_ = 0.965, RMSECV = 0.630%). The MicroNIR shows generally moderate performance; on PLSR, it exhibits worse performance than the NIR-S-G1, notably in grape seed extract (*R*^2^_CV_ = 0.767, RMSECV = 2.128%) and antioxidant capacity (R^2^_CV_ = 0.833, RMSECV = 0.528 µmol/g) analysis. However, the MicroNIR in none of these cases delivered a particularly inferior performance, showing a good all-around robustness.

On the other hand, the microPHAZIR tends to perform best for selected single constituent analysis like gallic (*R*^2^_CV_ = 0.738, RMSECV = 0.446 mg/g), catechin (*R*^2^_CV_ = 0.727, RMSECV = 2.030 mg/g), and epicatechin (*R*^2^_CV_ = 0.738, RMSECV = 0.446 mg/g), outperforming other handhelds in these cases. However, its predictive performance drops for procyanidin and caffeic acid. The microPHAZIR also tended to deliver a good performance for procyanidin and green tea extract content prediction, particularly with SVR models (respectively, *R*^2^_CV_ of 0.726, RMSECV of 3.753 mg/g, and *R*^2^_CV_ of 0.895, RMSECV of 1.092%). This seems to show a somewhat greater selectivity of this instrument towards specific chemical constituents. Given the narrowest wavelength region of microPHAZIR and the fact that the absorption bands of the bag material are manifested within it, this outcome is understandable. This suggests that its capability to detect and quantify specific constituents depends on the specific characteristic bands of these molecular structures located in the combination band region, provided the LDPE absorption bands do not interfere too greatly with the targeted analyte bands.

Regarding the differences between the two applied algorithms, the general consensus of previous research is that SVR, due to its tolerance to outliers and flexibility with different kernel functions, provides better predictive models for complex matrices compared to strictly linear algorithms, like PLSR [53,54,72]. Present findings, however, do not support the clear superiority of SVR in building predictive models based on these proanthocyanidin-rich extracts. While it produced marginally better models based on benchtop data in most cases, it was overall less feasible to build models based on handheld data. This could potentially be due to the bigger sample size requirement of SVR [83] to build reliable models and the observation that the correlation between spectral data and the predicted analyte is mostly linear in the current study. This was proposed by the superior performance of linear kernels for most SVR models. Some of the previous research compared these algorithms while investigating milk powder samples [53,84], a matrix known for much more complex vibrational bands and interactions due to the presence of various macromolecules. In this regard, the chemical composition of these extracts is simpler, mainly containing C-H and O-H groups and an occasional aromatic ring, which, therefore, does not necessitate the use of more sophisticated algorithms capable of dealing with non-linear patterns.

Apart from certain limitations of a feasibility study, these findings highlight the potential of benchtop NIR devices, covering the first and second overtone as well as combination band regions, in combination with PLSR or SVR, to reliably detect GSE adulteration and fortification while simultaneously quantifying key chemical parameters. Additionally, the results suggest that handheld devices, which capture most of the key spectral regions, can also predict these parameters decently. For this to be properly verified, the application of portable devices specifically designed for this region should be investigated. To improve robustness and facilitate industrial application, predictive models should include extracts of different plant variants to mitigate the effects of matrix dependency. While the present results do not warrant it, the application of more sophisticated non-linear predictive algorithms should also be considered, especially if the complexity of the matrix is to be further increased [65]. While the study focused more on the fingerprinting benefits of NIRS to quantify extracts, detailed chemical profiling and the identification of exclusive biomarkers in these compounds could still be valuable to produce more specific predictive models. The authors believe that the present study laid the groundwork for these developments and managed to take one step forward to a future where plant-based dietary supplements are safer and better characterized on the market.

## 5. Conclusions

Plant-based dietary supplements, such as grape seed extracts, are important commodities of the market not just because of their well-proven health-promoting benefits but because of their contribution to waste management and circular economy, as most of these compounds are derivatives of agri-food industry by-products. These products, despite their high economic value, are currently very poorly characterized on the market, where misclassification, batch-to-batch inconsistency, and economically motivated adulteration are all too common. One of the main issues behind this phenomenon is the lack of rapid, affordable and adequately selective quality control methods capable of reliably characterizing these commodities. The present research aimed at filling this gap by proposing for the first time an NIRS-based method using benchtop and multiple portable NIRS devices to characterize and quantitatively assess grape seed extract adulteration or fortification. During the chemical investigation, characteristic phenolic compounds were identified for each extract by combining wet chemistry results with exploratory NIR spectroscopic data analysis based on raw spectra and PCA. Univariate statistical analysis unveiled significant differences between the composition of the different extracts, whereas PLSR and SVR were applied as multivariate data analysis tools to build and optimize predictive models for each parameter and device combination. Models based on the benchtop device gave robust predictions with both algorithms, reaching a minimum *R*^2^_p_ value of 0.99 and maximum RMSEP of 0.27% for the prediction of all extract concentrations. Models based on handheld device data were comparably feasible to predict green tea extract concentration, procyanidin and caffeic acid content, whereas weaker performance was observed when predicting pine bark extract concentration and proanthocyanidin monomers. The present findings suggest that handheld devices covering the wavelength range between ~1400 and 2200 nm, identified as prominent for the analysis in focus here, should provide feasible results to predict most parameters that characterize these extracts. The study further verifies the applicability of handheld devices to assess the quality of dietary supplements by scanning directly through the packaging, which is a valuable benefit of the NIRS technique at various steps of production. The authors also propose further research investigating additional polyphenolic extracts (e.g., peanut skin extract) and different variants of the current ones while applying more sophisticated machine learning algorithms to build robust, industrially applicable predictive models. The current study has proven the relevance of (portable) NIR spectroscopy to better characterize plant-based extracts and could serve as the basis for future developments to improve the safety and transparency of the dietary supplement industry, which is in dire need of improving quality control schemes.

## Figures and Tables

**Table 1:** PLSR results for all instruments to predict extract concentrations. Error values are in %*w*/*w*.

	Grape Seed Extract Content				Pine Bark Extract Content				Green Tea Extract Content			
Instrument	NIRS XDS	NIR-S-G1	MicroNIR	MicroPHAZIR	NIRS XDS	NIR-S-G1	MicroNIR	MicroPHAZIR	NIRS XDS	NIR-S-G1	MicroNIR	MicroPHAZIR
Range (wl)	1100–2250	950–1650	950–1650	1630–2250	1100–2250	950–1650	950–1650	1630–2250	1100–2250	950–1650	950–1650	1630–2250
Pre-treat.	SG-31 + SNV	SG-21 + deTr	SG-11 + deTr + SNV	SG-11 + deTr + SNV	SG-21 + MSC	SG-31 + deTr + SNV	SG-21 + deTr + SNV	SG-11 + SNV	SG-21 + SNV	SG-11 + deTr	SG-21 + deTr + SNV	SG-11 + SNV
LV	7	8	12	9	7	12	14	10	4	8	12	6
RMSEC	0.393	2.037	1.769	1.929	0.234	1.822	1.704	1.580	0.394	0.550	0.795	0.982
*R* ^2^_C_	0.992	0.786	0.839	0.809	0.995	0.688	0.717	0.744	0.985	0.973	0.943	0.915
RMSECV	0.446	2.271	2.128	2.140	0.277	2.083	2.061	1.790	0.423	0.630	0.914	1.092
*R* ^2^_CV_	0.990	0.734	0.767	0.764	0.992	0.592	0.586	0.671	0.983	0.965	0.925	0.895

**Table 2:** SVR results for all instruments to predict extract concentrations. Error values are in %*w*/*w*.

	Grape Seed Extract Content				Pine Bark Extract Content				Green Tea Extract Content			
Instrument	NIRS XDS	NIR-S-G1	MicroNIR	MicroPHAZIR	NIRS XDS	NIR-S-G1	MicroNIR	MicroPHAZIR	NIRS XDS	NIR-S-G1	MicroNIR	MicroPHAZIR
Range (wl)	1100–2250	950–1650	950–1650	1630–2250	1100–2250	950–1650	950–1650	1630–2250	1100–2250	950–1650	950–1650	1630–2250
Pre-treat.	SG-31 + SNV	SG-21 + deTr	SG-11 + deTr + SNV	SG-11 + deTr + SNV	SG-21 + MSC	SG-31 + deTr + SNV	SG-21 + deTr + SNV	SG-11 + SNV	SG-21 + SNV	SG-11 + deTr	SG-21 + deTr + SNV	SG-11 + SNV
PC	7	23	8	17	12	14	8	17	13	16	26	17
Kernel	linear	linear	linear	linear	linear	linear	linear	linear	linear	linear	linear	linear
ε	0.1	0.5	0.1	0.01	0.01	0.5	0.5	0.5	0.01	0.1	0.1	0.5
Cost	10	1	0.25	110	10	0.24	0.4	10	10	10	10	1
RMSEC	0.512	2.131	2.659	2.772	0.233	2.186	2.262	2.231	0.374	0.561	0.809	1.565
*R* ^2^_C_	0.986	0.762	0.626	0.596	0.995	0.559	0.486	0.510	0.987	0.971	0.938	0.766
RMSECV	0.566	2.448	2.768	2.957	0.268	2.372	2.678	2.492	0.396	0.644	0.903	1.673
*R* ^2^_CV_	0.983	0.685	0.600	0.540	0.993	0.480	0.290	0.388	0.985	0.961	0.924	0.733

**Table 3:** PLSR results for all instruments to predict the procyanidin content (mg/g), antioxidant capacity (µmol/g) and caffeic acid content (mg/g) of the samples.

	Procyanidin Content				Antioxidant Capacity				Caffeic Acid Content			
Instrument	NIRS XDS	NIR-S-G1	MicroNIR	MicroPHAZIR	NIRS XDS	NIR-S-G1	MicroNIR	Micro- PHAZIR	NIRS XDS	NIR-S-G1	MicroNIR	MicroPHAZIR
Range (wl)	1100–2250	950–1650	950–1650	1630–2250	1100–2250	950–1650	950–1650	1630–2250	1100–2250	950–1650	950–1650	1630–2250
Pre-treat.	MSC	SG-25 + deTr + SNV	SG-21 + deTr + SNV	SG-11 + SNV	SG-21 + deTr + MSC	SG-21 + deTr + SNV	SG-11 + deTr + SNV	SG-11 + MSC	SG-21 + deTr + SNV	SNV	SG-21 + SNV	SG-11 + SNV
LV	4	12	11	6	3	4	6	10	4.000	8.000	14	10
RMSEC	0.948	1.329	2.167	2.165	0.202	0.379	0.478	0.400	0.018	0.026	0.031	0.039
*R* ^2^_C_	0.983	0.967	0.919	0.913	0.976	0.918	0.863	0.905	0.984	0.963	0.951	0.915
RMSECV	1.012	1.629	2.530	2.391	0.214	0.403	0.528	0.456	0.019	0.031	0.038	0.045
*R* ^2^_CV_	0.980	0.950	0.890	0.894	0.974	0.907	0.8331	0.877	0.982	0.951	0.929	0.890

**Table 4:** SVR results for all instruments to predict the procyanidin content (mg/g), antioxidant capacity (µmol/g) and caffeic acid content (mg/g) of the samples.

	Procyanidin Content				Antioxidant Capacity				Caffeic Acid Content			
Instrument	NIRS XDS	NIR-S-G1	MicroNIR	MicroPHAZIR	NIRS XDS	NIR-S-G1	MicroNIR	MicroPHAZIR	NIRS XDS	NIR-S-G1	MicroNIR	MicroPHAZIR
Range (wl)	1100–2250	950–1650	950–1650	1630–2250	1100–2250	950–1650	950–1650	1630–2250	1100–2250	950–1650	950–1650	1630–2250
Pre-treat.	MSC	SG-25 + deTr + SNV	SG-21 + deTr + SNV	SG-11 + SNV	SG-21 + deTr + MSC	SG-21 + deTr + SNV	SG-11 + deTr + SNV	SG-11 + MSC	SG-21 + deTr + SNV	SNV	SG-21 + SNV	SG-11 + SNV
PC	13	13	26	18	7	18	24	21	9	19	12	22
Kernel	linear	linear	linear	linear	linear	linear	linear	linear	linear	linear	linear	linear
ε	0.01	0.01	0.01	0.5	0.01	0.01	0.1	0.1	0.1	0.1	0.5	0.01
Cost	10	0.1	1	1	1	1	10	10	1	10	10	1
RMSEC	0.764	2.018	1.960	3.500	0.159	0.335	0.385	0.648	0.017	0.029	0.053	0.067
*R* ^2^_C_	0.989	0.922	0.925	0.761	0.984	0.930	0.907	0.737	0.985	0.957	0.854	0.764
RMSECV	0.805	2.331	2.206	3.753	0.167	0.373	0.436	0.704	0.019	0.034	0.058	0.070
*R* ^2^_CV_	0.988	0.896	0.907	0.726	0.983	0.914	0.883	0.690	0.982	0.938	0.823	0.737

**Table 5:** PLSR results for all instruments to predict the gallic acid (mg/g), catechin (mg/g) and epicatechin (mg/g) content of the samples.

	Gallic Acid Content				Catechin Content				Epicatechin Content			
Instrument	NIRS XDS	NIR-S-G1	MicroNIR	MicroPHAZIR	NIRS XDS	NIR-S-G1	MicroNIR	MicroPHAZIR	NIRS XDS	NIR-S-G1	MicroNIR	MicroPHAZIR
Range (wl)	1100–2250	950–1650	950–1650	1630–2250	1100–2250	950–1650	950–1650	1630–2250	1100–2250	950–1650	950–1650	1630–2250
Pre-treat.	SG-21 + deTr + SNV	SG-21 + deTr	SG-21 + deTr + MSC	SG-11 + SNV	SG-21 + deTr + SNV	SG-21 + FD	SG-21 + deTr + MSC	SG-11 + SNV	SG-21 + deTr + SNV	SG-21 + deTr	SG-21 + deTr + SNV	SG-11 + MSC
LV	6	9	11	10	6	15	15	11	6.000	15.000	15	15
RMSEC	0.609	2.680	2.947	2.447	0.318	1.798	1.983	1.807	0.086	0.401	0.426	0.369
*R* ^2^_C_	0.988	0.774	0.727	0.816	0.994	0.775	0.724	0.784	0.990	0.768	0.750	0.821
RMSECV	0.682	2.978	3.506	2.738	0.373	2.224	2.492	2.030	0.097	0.484	0.531	0.446
*R* ^2^_CV_	0.985	0.721	0.614	0.769	0.991	0.655	0.5634	0.727	0.987	0.663	0.610	0.738

**Table 6:** SVR results for all instruments to predict the gallic acid (mg/g), catechin (mg/g) and epicatechin (mg/g) content of the samples.

	Gallic Acid Content				Catechin Content				Epicatechin Content			
Instrument	NIRS XDS	NIR-S-G1	MicroNIR	MicroPHAZIR	NIRS XDS	NIR-S-G1	MicroNIR	MicroPHAZIR	NIRS XDS	NIR-S-G1	MicroNIR	MicroPHAZIR
Range (wl)	1100–2250	950–1650	950–1650	1630–2250	1100–2250	950–1650	950–1650	1630–2250	1100–2250	950–1650	950–1650	1630–2250
Pre-treat.	SG-21 + deTr + SNV	SG-21 + deTr	SG-21 + deTr + MSC	SG-11 + SNV	SG-21 + deTr + SNV	SG-21 + FD	SG-21 + deTr + MSC	SG-11 + SNV	SG-21 + deTr + SNV	SG-21 + deTr	SG-21 + deTr + SNV	SG-11 + MSC
PC	7	8	6	18	9	9	5	22	9	8	5	21
Kernel	linear	radial	linear	linear	linear	radial	radial	linear	linear	radial	radial	linear
ε	0.01	0.5	0.5	0.1	0.01	0.1	0.1	0.5	0.01	0.1	0.5	0.1
Cost	10	0.3	0.25	10	10	0.3	0.5	10	1	0.25	0.3	10
RMSEC	0.727	3.506	3.666	3.496	0.313	2.609	2.808	2.521	0.086	0.539	0.563	0.555
*R* ^2^_C_	0.983	0.613	0.574	0.614	0.994	0.546	0.466	0.578	0.990	0.605	0.566	0.582
RMSECV	0.809	3.696	3.852	3.764	0.360	3.248	3.447	2.816	0.093	0.662	0.682	0.608
*R* ^2^_CV_	0.979	0.570	0.529	0.553	0.991	0.297	0.195	0.473	0.988	0.404	0.363	0.499

## Data Availability

Data will be made available upon request.
